# Sex Differences in the Relationship Between Nutrient Intake and Mortality Results of the Shika Cohort Study

**DOI:** 10.3390/nu17050755

**Published:** 2025-02-21

**Authors:** Koichiro Hayashi, Masaharu Nakamura, Hiromasa Tsujiguchi, Akinori Hara, Keita Suzuki, Sakae Miyagi, Chie Takazawa, Jiaye Zhao, Jam Camara, Talica Marama, Atsushi Asai, Koji Katano, Tomoko Kasahara, Kuniko Sato, Aya Ogawa, Shinobu Fukushima, Aki Shibata, Fumihiko Suzuki, Yukari Shimizu, Yasuhiro Kambayashi, Takayuki Kannon, Yumie Takeshita, Hirohito Tsuboi, Atsushi Tajima, Tadashi Konoshita, Toshinari Takamura, Hiroyuki Nakamura

**Affiliations:** 1 Department of Public Health, Graduate School of Advanced Preventive Medical Sciences, Kanazawa University, 13-1 Takaramachi, Kanazawa 920-8640, Ishikawa, Japant-hiromasa@med.kanazawa-u.ac.jp (H.T.); hara-akinori@med.kanazawa-u.ac.jp (A.H.); 2 Department of Hygiene and Public Health, Faculty of Medicine, Institute of Medical, Pharmaceutical and Health Sciences, Kanazawa University, Kanazawa 920-8640, Ishikawa, Japanshinobu.19800715@gmail.com (S.F.); akintoki1116@gmail.com (A.S.);; 3 Advanced Preventive Medical Sciences Research Center, Kanazawa University, 13-1 Takaramachi, Kanazawa 920-8640, Ishikawa, Japanatajima@med.kanazawa-u.ac.jp (A.T.); 4 Innovative Clinical Research Center, Kanazawa University, 13-1 Takaramachi, Kanazawa 920-8641, Ishikawa, Japan; 5 Department of Clinical Cognitive Neuroscience, Graduate School of Medical Science, Kanazawa University, Kakuma-machi, Kanazawa 920-1192, Ishikawa, Japan; 6 Faculty of Nutrition, Osaka Seikei College, 3-10-62 Aikawa, Higashiyodogawa-ku, Osaka 533-0007, Osaka, Japan; a.ogawa523@gmail.com; 7 Department of Geriatric Dentistry, Ohu University School of Dentistry, 31-1 Misumido, Tomitamachi, Koriyama 963-8611, Fukushima, Japan; 8 Department of Nursing, Faculty of Health Sciences, Komatsu University, 14-1 He Mukai-Motoori-Machi, Komatsu 923-0961, Ishikawa, Japan; 9 Department of Public Health, Faculty of Veterinary Medicine, Okayama University of Science, 1-3 Ikoi-no-oka, Imabari 794-8555, Ehime, Japan; 10 Department of Biomedical Data Science, School of Medicine, Fujita Health University, 1-98 Dengaku-gakubo, Kutsukake-cho, Toyoake 470-1192, Aichi, Japan; 11 Department of Endocrinology and Metabolism, Graduate School of Medical Sciences, Kanazawa University, Kanazawa 920-8640, Ishikawa, Japan; 12 Graduate School of Human Sciences, The University of Shiga Prefecture, 2500 Hassaka-cho, Hikone 522-8533, Shiga, Japan; 13 Department of Bioinformatics and Genomics, Graduate School of Advanced Preventive Medical Sciences, Kanazawa University, 13-1 Takaramachi, Kanazawa 920-8640, Ishikawa, Japan; 14 Division of Diabetes Endocrinology and Metabolism, Yachiyo Medical Center, Tokyo Women’s Medical University, 477-96 Owada-Shinden, Yachiyo 276-8524, Chiba, Japan

**Keywords:** sex difference, nutrients intake, mortality

## Abstract

**Background/Objectives**: Macronutrients (protein, fat, and carbohydrate) provide sources of energy and play crucial roles in various physiological functions. However, sex differences in the relationships between protein, fat, and carbohydrate intakes and all-cause mortality remain unclear. The present study investigated sex differences in the relationships between protein, fat, and carbohydrate intakes and all-cause mortality using longitudinal epidemiological data. **Methods**: A total of 3743 healthy residents (1666 men and 2077 women) aged 40 years or older were followed up (men: 6.64 ± 1.51 years, women: 6.76 ± 1.28 years from 2013) in Shika Town, Ishikawa Prefecture, Japan. Nutrient intake was assessed at the baseline survey using the Brief Self-Administered Dietary History Questionnaire. The prospective relationship between baseline nutrient intake and all-cause mortality during the follow-up period, stratified by sex, was evaluated using two-way analysis of covariance and multiple logistic regression analysis, adjusted for age and BMI. **Results**: We documented 330 deaths (179 men and 151 women) during the 10-year follow-up period. Significant interactions between death and sex were observed for the intake of total protein (*p* < 0.001), animal protein (*p* < 0.001), vegetable protein (*p* = 0.033), total fat (*p* = 0.012), and animal fat (*p* = 0.024). Multiple logistic regression analysis demonstrated that total protein (*p* = 0.004), and animal protein (*p* = 0.010) decreased the all-cause mortality and increased carbohydrates (*p* = 0.046) in women. In men, total fat (*p* = 0.017) decreased the all-cause mortality. **Conclusions**: The present study revealed distinct sex differences in the effects of total protein, animal protein, and carbohydrate intakes on all-cause mortality. This sex difference may be due to the sex differences in nutrients intake themselves.

## 1. Introduction

Macronutrients (protein, fat, and carbohydrate) provide a source of energy and play crucial roles in various physiological functions [1]. Proteins are essential for tissue growth and repair, enzyme and hormone production, and immune function. Fats serve as a concentrated energy source, facilitate the absorption of fat-soluble vitamins, and contribute to cell membrane integrity. Carbohydrates are the body’s primary energy source, supporting brain function and physical activity. These macronutrients work together to maintain overall health and metabolic balance.

Previous studies examined the relationship between these nutrients and mortality. However, sex differences in the relationships between protein, fat, and carbohydrate intakes and all-cause mortality remain unclear. The Prospective Urban Rural Epidemiology (PURE) study reported that a high intake of carbohydrate and low intake of fat were associated with an increased risk of mortality [2]. This study was a large-scale multinational cohort study involving participants from 18 countries, with regions differing in their ranges of carbohydrate and fat intake. The study also suggested that different types of fat (saturated, monounsaturated, and polyunsaturated) had varying effects on health outcomes. These findings suggested that the effect of nutrient intake on mortality may vary by region. A cohort study that followed 42,192 Koreans reported that the range of a lower risk of mortality was 50–60% for carbohydrate and 30–40% for fat, while no correlation as observed between protein and mortality [3]. A U-shaped relationship between carbohydrate intake and mortality was reported in the Atherosclerosis Risk in Communities (ARIC) study [4]. However, a consensus on sex differences has not yet been reached. The Japan Multi-Institutional Collaborative Cohort (J-MICC) study, which included 34,893 men and 46,440 women, examined the relationship between nutrient intake and all-cause mortality based on longitudinal data and reported that a low carbohydrate intake was associated with high all-cause mortality in men [5]. The Japan Public Health Center (JPHC)-based prospective study, which examined the relationships between the intakes of total protein, vegetable protein, and animal protein and pneumonia mortality, found that a high total protein intake was associated with lower pneumonia mortality in women only [6]. Nagata et al. performed a prospective cohort study that followed 28,356 Japanese community residents and found that a higher fat intake was associated with lower all-cause mortality [7]. On the other hand, Wakai et al. reported a U-shaped relationship between fat intake and all-cause mortality in women only [8]. Based on these findings, further research is warranted on sex differences.

Shika town is a representative local community within Japan’s super-aged society. As of 2020, individuals aged 65 and older accounted for 44.7% of Shika’s total population [9]. In 2011, Kanazawa University and Shika town entered into a health promotion agreement, and since 2013, they have been conducting annual super preventive medical checkups. The Shika study has been reported in multiple articles over the years [10].

Therefore, participants were recruited from the Shika study cohort project conducted Shika town and followed them for 6.71 ± 1.39 years to investigate sex differences in the effects of protein, fat, and carbohydrate intakes on all-cause mortality using longitudinal data.

## 2. Materials and Methods

### 2.1. Subjects

**Figure 1:**
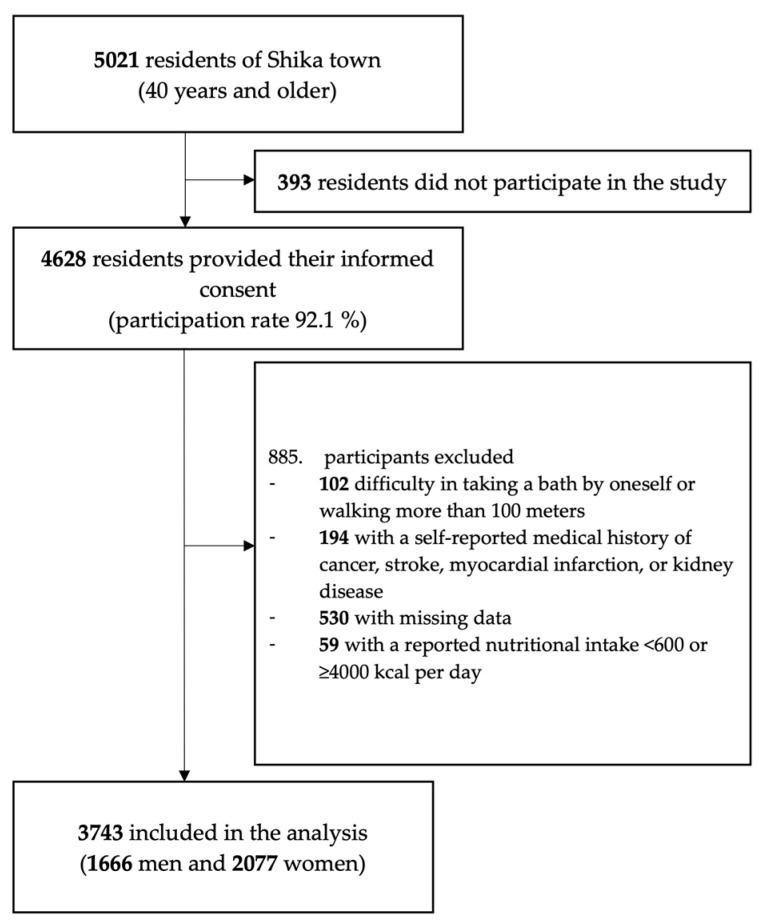
Flow chart of the study population.

All residents aged 40 years or older who had a certificate of residence in the model districts (Horimatsu, Higashi Masuho, Tsuchida, and Togi) of Shika town, Ishikawa Prefecture, Japan were included in this study. Shika town is located on the Noto Peninsula and had a total population of 22,663 in April 2013. A baseline survey was conducted in 2013–2016 to collect information on nutrient intake and participant characteristics. The baseline study included 5021 subjects, and 4628 participated in the study (participation rate, 92.1%). The participation criteria were as follows: ability to take a bath independently and walk at least 100 m as conditions for adequate activities of daily living (ADL). In addition, participants with cancer, stroke, myocardial infarction, kidney disease, missing nutrition data, or an energy intake <600 or ≥4000 kcal per day were excluded. Therefore, 3743 subjects (1666 men and 2077 women) were ultimately followed up (Figure 1).

This study received approval from the Kanazawa University Ethics Committee (protocol No. 1491; approved on 18 December 2013). Informed consent was obtained from all participants involved in the study.

### 2.2. Follow-Up Survey

In the follow-up survey, information on the survival status was collected between April 2013 and March 2023. We received monthly reports on the death of any participants from Shika town based on submitted death notifications.

The mean follow-up period was 6.64 ± 1.51 years for 1666 men (mean age, 62.84 ± 12.20 years) and 6.76 ± 1.28 years for 2077 women (mean age, 65.17 ± 13.08 years). The mean time from baseline to death was 3.87 ± 2.04 years for 179 men and 4.38 ± 1.93 years for 151 women. The mean age at death was 80.38 ± 9.60 years for men and 86.33 ± 8.55 years for women. Females were significantly older than males (*p* < 0.001).

### 2.3. Basic Demographics

Sex, age, height, weight, and ADL were obtained from the questionnaire. The body mass index (BMI) was calculated by dividing weight (kg) by the square of height (m^2^). Regarding ADL, subjects who responded “not at all difficult” from the following options: (1) very difficult, (2) a little difficult, and (3) not difficult at all, to the question on how difficult it is for them to “walk about 100 m” and “take a bath and change clothes by themselves” for health reasons were included in the follow-up sample. A question on medical history, namely, whether the subject had cancer, stroke, myocardial infarction, kidney disease, or was currently receiving treatment, was asked in the baseline survey.

### 2.4. Evaluation of Nutrient Intake

Nutrient intake was assessed with the Brief Self-Administered Dietary History Questionnaire (BDHQ). The BDHQ collects data on the average frequency of food intake and eating habits over the past month. It calculates the intake of 58 foods and beverages, as well as more than 100 nutrients, using a proprietary algorithm. These 58 foods and beverages are commonly eaten in Japan and are derived from the food list of the National Health and Nutrition Survey conducted by the Ministry of Health, Labor, and Welfare. The questionnaire covers seasonings, cooking oils, and various forms of food, including raw, cooked, processed, and pickled items. It also considers eating habits, such as the amount of noodle soup consumed and the intensity of seasoning. In this study, nutrient intake was adjusted using the density method. The reproducibility and validity of the BDHQ have already been established. Further details about the BDHQ can be found elsewhere [11,12,13].

### 2.5. Statistical Analysis

The unpaired Student’s *t*-test was employed to compare the means of continuous variables, while the chi-square test was used to compare the percentages of categorical variables. A two-way analysis of covariance adjusted for age and BMI was used to test interactions between all-cause mortality and sex for nutrient intake. The prospective relationship between baseline nutrient intake and all-cause mortality during follow-up, stratified by sex was assessed using multiple logistic regression analysis, adjusted for age and BMI. The analyses were conducted using version 25 of the Statistical Package for Social Sciences (IBM Corp., Tokyo, Japan). A significance level of *p* < 0.05 was applied to all analyses.

## 3. Results

### 3.1. Baseline Characteristics of Study Participants

Table 1 shows participant characteristics. During the follow-up period, there were 330 deaths (179 men and 151 women). All-cause mortality was significantly higher for men than for women (*p* < 0.001). Mean age in the baseline survey was 62.84 ± 12.20 years for males and 65.17 ± 13.08 years for females, with females being significantly older than males (*p* < 0.001). BMI (*p* < 0.001), the smoking status (*p* < 0.001), energy intake (*p* < 0.001), and alcohol intake (*p* < 0.001) were significantly higher in men than in women.

The percentage of participants living alone (*p* < 0.001) and protein (*p* < 0.001), animal protein (*p* < 0.001), vegetable protein (*p* < 0.001), fat (*p* < 0.001), animal fat (*p* < 0.001), vegetable fat (*p* < 0.001), SFA (*p* < 0.001), MUFA (*p* < 0.001), PUFA (*p* < 0.001), *n*-3 fatty acid (*p* < 0.001), *n*-6 fatty acid (*p* < 0.001), and carbohydrate (*p* < 0.001) intakes were significantly higher in women than in men.

### 3.2. Comparison of Subjects by Sex and Mortality

Table 2 shows a comparison of participants stratified by sex and mortality. Regarding mean age in the baseline survey, the mean age of men was 61.20 ± 11.43 years in the non-event group and 76.51 ± 9.48 years in the event group. The event group was significantly older than the non-event group (*p* < 0.001). The mean age of women was 63.85 ± 12.46 years in the non-event group and 81.95 ± 8.43 years in the event group. The event group was significantly older than the non-event group (*p* < 0.001). The mean age at death was 80.38 ± 9.60 years for males and 86.33 ± 8.55 years for females. The mean follow-up period in men was 6.98 ± 1.00 years in the non-event group and 3.87 ± 2.04 years in the event group, which was significantly longer in the non-event group (*p* < 0.001). The mean follow-up period in women was significantly longer in the non-event group (6.76 ± 1.28 years) than in the event group (4.38 ± 1.93 years) (*p* < 0.001).

Regarding nutrient intake, energy intake was not associated with mortality for both sexes. In men, protein (*p* = 0.005), vegetable protein (*p* = 0.002), and carbohydrate (*p* = 0.001) intakes were significantly higher in the event group than in the non-event group. In contrast, SFA (*p* = 0.032), MUFA (*p* = 0.007), *n*-6 fatty acid (*p* = 0.007), and alcohol (*p* < 0.001) intakes were significantly lower in the event group than in the non-event group. In women, protein (*p* < 0.001), animal protein (*p* < 0.001), lipid (*p* < 0.001), animal fat (*p* < 0.001), vegetable fat (*p* = 0.002), SFA (*p* < 0.001), MUFA (*p* < 0.001), PUFA (*p* < 0.001), *n*-3 fatty acid (*p* < 0.001), *n*-6 fatty acid (*p* < 0.001), and alcohol (*p* = 0.016) intakes were significantly higher in the non-event group than in the event group. Conversely, carbohydrate intake (*p* < 0.001) was significantly lower in the non-event group than in the event group.

### 3.3. Interactions Between Sex and Mortality for Nutrient Intake

Table 3 displays the interactions between sex and mortality for nutrient intake. There was a main effect of mortality on the mean intakes of protein (*p* < 0.001), animal protein (*p* < 0.001), total fat (*p* = 0.003), animal fat (*p* = 0.008), SFA (*p* = 0.016), MUFA (*p* = 0.014), PUFA (*p* = 0.005), *n*-3 fatty acids (*p* < 0.001), *n*-6 fatty acids (*p* = 0.022), and carbohydrate (*p* < 0.001). The main effect of sex was observed for the mean intakes of vegetable protein (*p* < 0.001), total fat (*p* < 0.001), animal fat (*p* = 0.006), vegetable fat (*p* < 0.001), SFA (*p* < 0.001), MUFA (*p* < 0.001), PUFA (*p* < 0.001), *n*-6 fatty acids (*p* < 0.001), and carbohydrate (*p* < 0.001).

Interactions between sex and mortality were observed for protein (*p* < 0.001), animal protein (*p* < 0.001), vegetable protein (*p* = 0.033), fat (*p* = 0.012), animal fat (*p* = 0.024), PUFA (*p* = 0.007), and *n*-3 fatty acids (*p* < 0.001).

### 3.4. Prospective Relationship Between Nutrient Intake and Mortality Stratified by Sex

Table 4 demonstrates the prospective relationship between nutrient intake and mortality according to sex. Negative correlations were observed between the following nutrient intakes and mortality in women: total protein (OR: 0.916; 95%CI: 0.862–0.973; *p* = 0.004), animal protein (OR: 0.927; 95%CI: 0.876–0.982; *p* = 0.010), PUFA (OR: 0.875; 95%CI: 0.773–0.991; *p* = 0.036), and *n*-3 fatty acids (OR: 0.621; 95%CI: 0.410–0.940; *p* = 0.024), while a positive correlation was observed between carbohydrates and mortality (OR: 1.024; 95%CI: 1.000–1.049; *p* = 0.046). In men, negative correlations were observed between the following nutrient intakes and mortality: total fat (OR: 0.963; 95%CI: 0.934–0.993; *p* = 0.017), SFA (OR: 0.866; 95%CI: 0.783–0.957; *p* = 0.005), MUFA (OR: 0.909; 95%CI: 0.839–0.984; *p* = 0.019), and *n*-6 fatty acids (OR: 0.854; 95%CI: 0.734–0.994; *p* = 0.042).

Therefore, the intakes of total protein (*p* = 0.004), animal protein (*p* = 0.010), PUFA (*p* = 0.036), and *n*-3 fatty acids (*p* = 0.024) decreased all-cause mortality in women. In contrast, carbohydrate intake increased all-cause mortality in women (*p* = 0.046). The intakes of total fat (*p* = 0.017), SFA (*p* = 0.005), MUFA (*p* = 0.019), and *n*-6 fatty acids (*p* = 0.042) decreased all-cause mortality in men.

## 4. Discussion

The present study examined the prospective relationship between nutrient intake and all-cause mortality by sex. The results obtained showed that high intakes of protein, animal protein, PUFA, and *n*-3 fatty acids decreased all-cause mortality, whereas a high carbohydrate intake increased all-cause mortality. High intakes of fat, SFA, MUFA, and *n*-6 fatty acids decreased all-cause mortality in men. Sex differences were observed in the prospective relationship between nutrient intake and all-cause mortality. These results suggest that in women, a low excess intake of carbohydrates and adequate intake of foods rich in protein (especially animal protein), PUFA, and *n*-3 fatty acids, such as bluefish, may contribute to a reduction in all-cause mortality. Among men, the findings indicate that the consumption of SFA-rich foods, such as meat and cheese, and nuts with high MUFA and *n*-6 fatty acid content, might also contribute to a decrease in all-cause mortality.

In this study, the energy intake of men was significantly higher than that of women. This result was consistent with the findings of the National Health and Nutrition Survey conducted by the Ministry of Health, Labor, and Welfare of Japan [14]. Among men, although the difference was not statistically significant, energy intake was lower in the event group than in the non-event group. In contrast, no significant difference in energy intake was observed between the event and non-event groups among women. Both men and women in the event group had lower fat intake and higher carbohydrate intake than those in the non-event group. However, protein intake showed a sex-specific pattern: men in the event group had a higher protein intake, whereas women had a lower intake, particularly of animal protein. This difference in protein intake may have contributed to the observed sex differences in total energy intake.

A meta-analysis of 31 studies revealed a decreased risk of all-cause mortality with a higher total protein intake as well as a decreased risk of all-cause mortality and cardiovascular disease mortality with a higher plant protein intake, while no correlation was found between animal protein and all-cause, cancer, or cardiovascular disease mortality [15]. In the present study, protein intake was associated with a lower risk of all-cause mortality in women only, which is consistent with the findings of the meta-analysis. However, no significant difference was observed between plant protein intake and all-cause mortality in women, whereas animal protein intake correlated with all-cause mortality. A higher animal protein intake was associated with lower all-cause mortality, which was not consistent with the findings of the meta-analysis. Katagiri et al. investigated the relationship between total protein, vegetable protein, and animal protein intakes and pneumonia mortality, a major cause of death in Japan, and reported that a higher total protein intake was associated with lower pneumonia mortality in women [6]. Similar results were obtained in the present study, with a significant difference being observed between protein intake and all-cause mortality in women only. One possible reason for these discrepancies could be differences in dietary patterns, overall nutritional intake, and lifestyle factors such as physical activity among study populations. Future research incorporating more detailed dietary assessments and lifestyle evaluations may help clarify these inconsistencies. The effects of lifestyle habits, such as drinking and smoking, may be one of the reasons why significant differences were not observed in men. According to the findings of the National Health and Nutrition Survey conducted by the Ministry of Health, Labor, and Welfare of Japan, a larger percentage of men than women consume alcohol in quantities that increase the risk of lifestyle-related diseases, while a higher percentage of men than women have a smoking habit [14]. Therefore, the effects of lifestyle may be stronger than those of protein intake. Further research is needed to clarify this issue. In the present study, higher total protein and animal protein intakes were associated with a lower risk of all-cause mortality in women; however, the underlying mechanisms remain unclear. Previous studies reported the importance of protein intake for preventing frailty among the elderly [16,17,18]. Moreover, nutritional support, including adequate protein intake, is important for the prevention and improvement of the “cold and dry” phenotype (Nohria-Stevenson classification) in elderly patients, which is characterized by poor nutritional status, frailty, and worse outcomes [19,20,21]. However, this appears to be dependent on the main source of animal protein, namely, red meat, such as beef or pork, or fish. A prospective cohort study by Haghighatdoost et al. showed that the risk of all-cause mortality was reduced by 21% with a higher fish intake [22]. Furthermore, in a pooled analysis of six prospective cohort studies, Zhong et al. demonstrated that substituting eggs, processed meat, unprocessed red meat, or poultry with nuts, whole grains, legumes, or fish reduced the risks of incident cardiovascular disease and all-cause mortality [23]. Therefore, it is important to not only classify protein intake into animal and plant sources, but also to consider the sources of animal protein. Although we did not analyze the sources of animal protein intake, we speculate that women had a higher intake of fish based on the present results showing that high intakes of PUFA and *n*-3 fatty acids inversely correlated with all-cause mortality.

The results of our multiple logistic regression analysis revealed that high intakes of total fat, SFA, MUFA, and *n*-6 fatty acids in men and PUFA and *n*-3 fatty acids in women negatively correlated with all-cause mortality. A meta-analysis by Jiang et al. showed that higher concentrations of marine-derived omega-3 PUFA biomarkers were associated with a significantly reduced risk of cardiovascular disease and all-cause mortality [24], which supports the present results on women. Regarding men, Nagata et al. found that all-cause mortality decreased with a higher fat intake in men only [7], which is also consistent with the present results. A meta-analysis, combined with the findings of cohort studies, examined the relationships between SFA intake and various health outcomes, including all-cause mortality, cardiovascular disease mortality, coronary artery disease mortality, and the incidence of coronary artery disease, stroke, and type 2 diabetes, and found no correlations in any of these outcomes [25]. Muto et al. conducted a meta-analysis and reported a negative relationship between SFA intake and the incidence of cerebral hemorrhage and stroke in Japanese individuals, in contrast to that in other nationalities [26], which is in accordance with the present results. In addition, a meta-analysis of 29 prospective cohort studies conducted by Mazidi et al. reported that total fat, PUFA, and MUFA intakes were inversely associated with all-cause mortality [27]. Conversely, a pooled analysis of the findings of a cohort study on the effects of replacing SFA with PUFA on the incidence of coronary artery disease reported a significant reduction in its incidence [28]. Therefore, the relationship between fat intake and mortality has not yet been established. Further detailed analyses, such as a longer follow-up period, are needed.

The present results revealed a correlation between a high carbohydrate intake and high all-cause mortality in women only. Seidelmann et al. reported that the lowest mortality rate was observed when carbohydrates accounted for 50–55% of energy intake, while the risk of death was higher for a carbohydrate intake lower than 40% and higher than 70% [4]. Tamura et al. showed that the risk of death in men was higher with a carbohydrate intake <40% than >50–55%, while mortality in women was higher with a carbohydrate intake >65% than 50–55% [5]. The present study found that a higher carbohydrate intake in women was associated with higher all-cause mortality, which is consistent with previous findings.

Although carbohydrate intake was significantly higher in the event group (55.85% ± 9.74) than in the non-event group (53.38% ± 9.41) (*p* = 0.001), carbohydrate intake did not correlate all-cause mortality in men. The difference in carbohydrate intake between the two groups did not appear to be large enough to affect all-cause mortality. Therefore, one possible explanation for this sex difference in the effects of carbohydrate intake on all-cause mortality was sex differences in the intake of the nutrient itself.

The strengths of the present study are as follows. The study design was longitudinal, which allowed us to show a prospective causal relationship between nutrient intake and all-cause mortality. Furthermore, the high follow-up rate minimized the selection bias (80.9% (3743 follow-up subjects/4628 participants)). In addition, we used a reliable questionnaire to estimate nutrient intake. These strengths enhance the validity and reliability of our findings. However, this study has several limitations. It was conducted in a single Japanese town with self-reported data, including nutritional intake and baseline health status. The ADL assessment was limited to walking and bathing, excluding other aspects. Additionally, potential confounding factors such as smoking, alcohol consumption, exercise levels, and causes of death were not assessed. Furthermore, as this study reflects the characteristics of a specific Japanese population, its findings may not be directly applicable to other regions or global populations.

## 5. Conclusions

The present study showed distinct sex differences in the effects of total protein, animal protein, MUFA, SFA, *n*-3 fatty acid, *n*-6 fatty acid, and carbohydrate intakes on all-cause mortality. These differences may result from variations in metabolism, hormonal effects, and lifestyle factors between males and females. Understanding these sex differences can help in developing more tailored dietary guidance, ultimately contributing to improved population health.

## Figures and Tables

**Table 1:** Baseline characteristics of study participants.

		All (*n* = 3743)				Males (*n* = 1666)				Females (*n* = 2077)				*p*-Value *
		Mean (*n*)		SD (%)		Mean (*n*)		SD (%)		Mean (*n*)		SD (%)		
Death, *n* (%)		330	(	8.82	)	179	(	10.74	)	151	(	7.27	)	<0.001
Age		64.13	±	12.75		62.84	±	12.20		65.17	±	13.08		<0.001
BMI, kg/m^2^		23.02	±	3.30		23.59	±	3.11		22.56	±	3.38		<0.001
Smoking, *n* (%)														<0.001
	Current	608	(	19.11	)	494	(	34.99	)	114	(	6.44	)	
	Ex	728	(	22.88	)	597	(	42.28	)	131	(	7.40	)	
	Non	1846	(	58.01	)	321	(	22.73	)	1525	(	86.16	)	
Education, *n* (%)														0.082
	Junior high school	1025	(	32.82	)	416	(	30.19	)	609	(	34.9	)	
	High school	1224	(	39.19	)	553	(	40.13	)	671	(	38.5	)	
	Junior college	539	(	17.26	)	155	(	11.25	)	305	(	17.5	)	
	University or higher	335	(	10.73	)	254	(	18.43	)	160	(	9.2	)	
Living alone, *n* (%)		303	(	9.42	)	111	(	7.79	)	192	(	10.7	)	<0.001
Nutrition														
Energy, kcal		1801.17	±	595.44		1995.12	±	623.93		1645.61	±	521.98		<0.001
Protein, % energy		15.22	±	3.27		14.59	±	3.26		15.73	±	3.18		<0.001
Animal protein, % energy		8.72	±	3.35		8.37	±	3.35		8.99	±	3.33		<0.001
Vegetable protein, % energy		6.51	±	1.14		6.21	±	1.13		6.74	±	1.09		<0.001
Fat, % energy		24.78	±	6.10		23.26	±	6.08		25.99	±	5.84		<0.001
Animal fat, % energy		11.70	±	4.24		11.11	±	4.16		12.17	±	4.24		<0.001
Vegetable fat, % energy		13.08	±	3.86		12.15	±	3.76		13.82	±	3.77		<0.001
SFA, % energy		6.59	±	1.96		6.05	±	1.88		7.02	±	1.92		<0.001
MUFA, % energy		8.72	±	2.38		8.24	±	2.38		9.11	±	2.32		<0.001
PUFA, % energy		6.11	±	1.57		5.83	±	1.58		6.33	±	1.52		<0.001
*n*-3 fatty acids, % energy		1.33	±	0.46		1.28	±	0.47		1.37	±	0.45		<0.001
*n*-6 fatty acids, % energy		4.75	±	1.26		4.53	±	1.26		4.93	±	1.23		<0.001
Carbohydrate, % energy		54.79	±	8.86		53.64	±	9.47		55.72	±	8.22		<0.001
Alcohol, % energy		3.74	±	6.77		6.79	±	8.29		1.30	±	3.74		<0.001

* Continuous variables were examined by an unpaired *t*-test and categorical variables by the chi-square test. Abbreviations: SFA, saturated fatty acid; MUFA, monounsaturated fatty acid; PUFA, polyunsaturated fatty acid.

**Table 2:** Comparison of subjects by sex and mortality.

		Males (*n* = 1666)									Females (*n* = 2077)								
		Non-Event (*n* = 1487)				Event (*n* = 179)				*p*-value *	Non-Event (*n* = 1926)				Event (*n* = 151)				*p*-Value *
		Mean (*n*)		SD (%)		Mean (*n*)		SD (%)			Mean (*n*)		SD (%)		Mean (*n*)		SD (%)		
Age (at baseline)		61.20	±	11.43		76.51	±	9.48		<0.001	63.85	±	12.46		81.95	±	8.43		<0.001
Age (at death)		-	±	-		80.38	±	9.60		-	-	±	-		86.33	±	8.55		-
Follow-up period		6.98	±	1.00		3.87	±	2.04		<0.001	6.94	±	1.00		4.38	±	1.93		<0.001
BMI, kg/m^2^		23.72	±	3.08		22.47	±	3.14		<0.001	22.62	±	3.37		21.79	±	3.44		0.005
Smoking, *n* (%)										<0.001									0.010
	Current	469	(	36.61	)	25	(	19.08	)		112	(	6.72	)	2	(	1.94	)	
	Ex	532	(	41.53	)	65	(	49.62	)		128	(	7.68	)	3	(	2.91	)	
	Non	280	(	21.86	)	41	(	31.30	)		1427	(	85.60	)	98	(	95.15	)	
Education, *n* (%)										0.006									0.002
	Junior high school	346	(	27.61	)	70	(	56.0	)		538	(	32.67	)	71	(	72.4	)	
	High school	519	(	41.42	)	34	(	27.2	)		650	(	39.47	)	21	(	21.4	)	
	Junior college	144	(	11.49	)	11	(	8.8	)		300	(	18.21	)	5	(	5.1	)	
	University or higher	244	(	19.47	)	10	(	8.0	)		159	(	9.65	)	1	(	1.0	)	
Living alone, *n* (%)		98	(	7.62	)	13	(	9.4	)	0.094	173	(	10.25	)	19	(	18.3	)	<0.001
Nutrition																			
Energy, kcal		2004.68	±	622.37		1915.64	±	632.95		0.076	1648.90	±	515.82		1603.64	±	595.32		0.365
Protein, % energy		14.50	±	3.23		15.27	±	3.45		0.005	15.82	±	3.17		14.70	±	3.13		<0.001
Animal protein, % energy		8.32	±	3.32		8.81	±	3.56		0.079	9.08	±	3.33		7.92	±	3.20		<0.001
Vegetable protein, % energy		6.18	±	1.13		6.46	±	1.14		0.002	6.74	±	1.10		6.78	±	1.05		0.663
Fat, % energy		23.36	±	6.08		22.47	±	6.02		0.062	26.19	±	5.77		23.47	±	6.13		<0.001
Animal fat, % energy		11.16	±	4.17		10.68	±	4.09		0.137	12.29	±	4.21		10.68	±	4.43		<0.001
Vegetable fat, % energy		12.20	±	3.75		11.79	±	3.82		0.175	13.90	±	3.73		12.79	±	4.17		0.002
SFA, % energy		6.09	±	1.90		5.80	±	1.67		0.032	7.07	±	1.91		6.42	±	1.97		<0.001
MUFA, % energy		8.30	±	2.38		7.80	±	2.31		0.007	9.18	±	2.30		8.10	±	2.36		<0.001
PUFA, % energy		5.85	±	1.58		5.66	±	1.59		0.120	6.38	±	1.50		5.68	±	1.55		<0.001
*n*-3 fatty acids, % energy		1.27	±	0.46		1.34	±	0.55		0.123	1.38	±	0.45		1.24	±	0.44		<0.001
*n*-6 fatty acids, % energy		4.56	±	1.26		4.29	±	1.23		0.007	4.97	±	1.22		4.42	±	1.25		<0.001
Carbohydrate, % energy		53.38	±	9.41		55.85	±	9.74		0.001	55.39	±	8.15		59.83	±	8.01		<0.001
Alcohol, % energy		7.03	±	8.34		4.77	±	7.58		<0.001	1.36	±	3.75		0.61	±	3.59		0.016

* Continuous variables were examined by the unpaired *t*-test and categorical variables by the chi-square test. Abbreviations: SFA, saturated fatty acid; MUFA, monounsaturated fatty acid; PUFA, polyunsaturated fatty acid.

**Table 3:** Interactions between sex and mortality for nutrient intake.

	All (*n* = 3743)										
	Males (*n* = 1666)				Females (*n* = 2077)				*p*-Value		
	Non-Event (*n* = 1487)		Event (*n* = 179)		Non-Event (*n* = 1926)		Event (*n* = 151)		Mortality *	Sex *	Mortality × Sex *
	Mean	SD	Mean	SD	Mean	SD	Mean	SD			
Protein, % energy	14.50	3.23	15.27	3.45	15.82	3.17	14.70	3.13	<0.001	0.285	<0.001
Animal protein, % energy	8.32	3.32	8.81	3.56	9.08	3.33	7.92	3.20	<0.001	0.389	<0.001
Vegetable protein, % energy	6.18	1.13	6.46	1.14	6.74	1.10	6.78	1.05	0.142	<0.001	0.033
Fat, % energy	23.36	6.08	22.47	6.02	26.19	5.77	23.47	6.13	0.003	<0.001	0.012
Animal fat, % energy	11.16	4.17	10.68	4.09	12.29	4.21	10.68	4.43	0.008	0.006	0.024
Vegetable fat, % energy	12.20	3.75	11.79	3.82	13.90	3.73	12.79	4.17	0.092	<0.001	0.150
SFA, % energy	6.09	1.90	5.80	1.67	7.07	1.91	6.42	1.97	0.016	<0.001	0.125
MUFA, % energy	8.30	2.38	7.80	2.31	9.18	2.30	8.10	2.36	0.014	<0.001	0.054
PUFA, % energy	5.85	1.58	5.66	1.59	6.38	1.50	5.68	1.55	0.005	<0.001	0.007
*n*-3 fatty acids, % energy	1.27	0.46	1.34	0.55	1.38	0.45	1.24	0.44	<0.001	0.774	<0.001
*n*-6 fatty acids, % energy	4.56	1.26	4.29	1.23	4.97	1.22	4.42	1.25	0.022	<0.001	0.073
Carbohydrate, % energy	53.38	9.41	55.85	9.74	55.39	8.15	59.83	8.01	<0.001	<0.001	0.091

* A two-way analysis of covariance (two-way ANCOVA). Covariates were adjusted for age and BMI. Abbreviations: SFA, saturated fatty acid; MUFA, monounsaturated fatty acid; PUFA, polyunsaturated fatty acid.

**Table 4:** Prospective relationship between nutrient intake and mortality stratified by sex.

		Odds Ratio	Lower 95% Confidence Limit	Upper 95% Confidence Limit	*p*-Value *
Females	Protein, % energy	0.916	0.862	0.973	0.004
(*n* = 2077)	Animal protein, % energy	0.927	0.876	0.982	0.010
	Vegetable protein, % energy	0.959	0.800	1.150	0.652
	Fat, % energy	0.973	0.943	1.005	0.098
	Animal fat, % energy	0.980	0.937	1.026	0.385
	Vegetable fat, % energy	0.963	0.917	1.010	0.121
	SFA, % energy	0.973	0.880	1.075	0.585
	MUFA, % energy	0.932	0.858	1.012	0.095
	PUFA, % energy	0.875	0.773	0.991	0.036
	*n*-3 fatty acids, % energy	0.621	0.410	0.940	0.024
	*n*-6 fatty acids, % energy	0.871	0.746	1.018	0.082
	Carbohydrate, % energy	1.024	1.000	1.049	0.046
Males	Protein, % energy	0.987	0.934	1.044	0.655
(*n* = 1666)	Animal protein, % energy	0.997	0.947	1.050	0.911
	Vegetable protein, % energy	0.928	0.793	1.086	0.350
	Fat, % energy	0.963	0.934	0.993	0.017
	Animal fat, % energy	0.960	0.918	1.004	0.072
	Vegetable fat, % energy	0.954	0.907	1.003	0.064
	SFA, % energy	0.866	0.783	0.957	0.005
	MUFA, % energy	0.909	0.839	0.984	0.019
	PUFA, % energy	0.912	0.811	1.026	0.126
	*n*-3 fatty acids, % energy	1.036	0.722	1.488	0.847
	*n*-6 fatty acids, % energy	0.854	0.734	0.994	0.042
	Carbohydrate, % energy	1.013	0.993	1.033	0.197

* A multiple logistic regression analysis adjusted for age and BMI. Abbreviations: SFA, saturated fatty acid; MUFA, monounsaturated fatty acid; PUFA, polyunsaturated fatty acid.

## Data Availability

The data described in the manuscript are available upon request from the corresponding author. The data are not publicly available due to privacy and ethical policies.

## Abbreviations

The following abbreviations are used in this manuscript:

ADL	Activities of Daily Living
BMI	Body Mass Index
BDHQ	Brief Self-Administered Dietary History Questionnaire
SFA	Saturated Fatty Acid
MUFA	Monounsaturated Fatty Acid
PUFA	Polyunsaturated Fatty Acid
SD	Standard Deviation
CI	Confidence Interval
OR	Odds Ratio
ANCOVA	Analysis of Covariance

## References

[1] Carreiro A.L., Dhillon J., Gordon S., Higgins K.A., Jacobs A.G., McArthur B.M., Redan B.W., Rivera R.L., Schmidt L.R., Mattes R.D. (2016). The Macronutrients, Appetite, and Energy Intake. Annu. Rev. Nutr..

[2] Dehghan M., Mente A., Zhang X., Swaminathan S., Li W., Mohan V., Iqbal R., Kumar R., Wentzel-Viljoen E., Rosengren A. (2017). Associations of Fats and Carbohydrate Intake with Cardiovascular Disease and Mortality in 18 Countries from Five Continents (PURE): A Prospective Cohort Study. Lancet.

[3] Kwon Y.J., Lee H.S., Park J.Y., Lee J.W. (2020). Associating Intake Proportion of Carbohydrate, Fat, and Protein with All-Cause Mortality in Korean Adults. Nutrients.

[4] Seidelmann S.B., Claggett B., Cheng S., Henglin M., Shah A., Steffen L.M., Folsom A.R., Rimm E.B., Willett W.C., Solomon S.D. (2018). Dietary Carbohydrate Intake and Mortality: A Prospective Cohort Study and Meta-Analysis. Lancet Public Health.

[5] Tamura T., Wakai K., Kato Y., Tamada Y., Kubo Y., Okada R., Nagayoshi M., Hishida A., Imaeda N., Goto C. (2023). Dietary Carbohydrate and Fat Intakes and Risk of Mortality in the Japanese Population: The Japan Multi-Institutional Collaborative Cohort Study. J. Nutr..

[6] Katagiri R., Yamaji T., Sawada N., Iwasaki M., Inoue M., Tsugane S. (2022). Total, Animal, and Plant Protein Intake and Pneumonia Mortality in the Japan Public Health Center-Based Prospective Study. Am. J. Clin. Nutr..

[7] Nagata C., Nakamura K., Wada K., Oba S., Tsuji M., Tamai Y., Kawachi T. (2012). Total Fat Intake Is Associated with Decreased Mortality in Japanese Men but Not in Women. J. Nutr..

[8] Wakai K., Naito M., Date C., Iso H., Tamakoshi A. (2014). Dietary Intakes of Fat and Total Mortality among Japanese Populations with a Low Fat Intake: The Japan Collaborative Cohort (JACC) Study. Nutr. Metab..

[9] Japan’s Regional Future Population Projections (2023 Estimate). https://www.ipss.go.jp/pp-shicyoson/j/shicyoson23/t-page.asp.

[10] Project S.H.I.P (Shikamachi Health Improvement Practice). http://www.projectship.org/achievement.html.

[11] Kobayashi S., Honda S., Murakami K., Sasaki S., Okubo H., Hirota N., Notsu A., Fukui M., Date C. (2012). Both Comprehensive and Brief Self-Administered Diet History Questionnaires Satisfactorily Rank Nutrient Intakes in Japanese Adults. J. Epidemiol..

[12] Kobayashi S., Murakami K., Sasaki S., Okubo H., Hirota N., Notsu A., Fukui M., Date C. (2011). Comparison of Relative Validity of Food Group Intakes Estimated by Comprehensive and Brief-Type Self-Administered Diet History Questionnaires against 16 d Dietary Records in Japanese Adults. Public Health Nutr..

[13] Takayama M., Arai Y., Sasaki S., Hashimoto M., Shimizu K., Abe Y., Hirose N. (2013). Association of marine-origin n-3 polyunsaturated fatty acids consumption and functional mobility in the community-dwelling oldest old. J. Nutr. Health Aging.

[14] 2019 National Health and Nutrition Survey Report. https://www.mhlw.go.jp/stf/seisakunitsuite/bunya/kenkou_iryou/kenkou/eiyou/r1-houkoku_00002.html.

[15] Naghshi S., Sadeghi O., Willett W.C., Esmaillzadeh A. (2020). Dietary Intake of Total, Animal, and Plant Proteins and Risk of All Cause, Cardiovascular, and Cancer Mortality: Systematic Review and Dose-Response Meta-Analysis of Prospective Cohort Studies. BMJ.

[16] Coelho-Junior H.J., Calvani R., Picca A., Tosato M., Landi F., Marzetti E. (2022). Protein Intake and Frailty in Older Adults: A Systematic Review and Meta-Analysis of Observational Studies. Nutrients.

[17] Rahi B., Colombet Z., Gonzalez-Colaço Harmand M., Dartigues J.F., Boirie Y., Letenneur L., Feart C. (2016). Higher Protein but Not Energy Intake Is Associated With a Lower Prevalence of Frailty Among Community-Dwelling Older Adults in the French Three-City Cohort. J. Am. Med. Dir. Assoc..

[18] Kobayashi S., Asakura K., Suga H., Sasaki S. (2013). High Protein Intake Is Associated with Low Prevalence of Frailty among Old Japanese Women: A Multicenter Cross-Sectional Study. Nutr. J..

[19] Nohria A., Tsang S.W., Fang J.C., Lewis E.F., Jarcho J.A., Mudge G.H., Stevenson L.W. (2003). Clinical assessment identifies hemodynamic profiles that predict outcomes in patients admitted with heart failure. J. Am. Coll. Cardiol..

[20] Ponikowski P., Voors A.A., Anker S.D., Bueno H., Cleland J.G.F., Coats A.J.S., Falk V., González-Juanatey J.R., Harjola V.P., Jankowska E.A. (2016). 2016 ESC Guidelines for the diagnosis and treatment of acute and chronic heart failure: The Task Force for the diagnosis and treatment of acute and chronic heart failure of the European Society of Cardiology (ESC). Developed with the special contribution of the Heart Failure Association (HFA) of the ESC. Eur. J. Heart Fail..

[21] Sonaglioni A., Lonati C., Tescaro L., Nicolosi G.L., Proietti M., Lombardo M., Harari S. (2022). Prevalence and clinical outcome of main echocardiographic and hemodynamic heart failure phenotypes in a population of hospitalized patients 70 years old and older. Aging Clin. Exp. Res..

[22] Haghighatdoost F., Mohammadifard N., Zakeri P., Najafian J., Sadeghi M., Roohafza H., Sarrafzadegan N. (2023). Differences in All-Cause Mortality Risk Associated with Animal and Plant Dietary Protein Sources Consumption. Sci. Rep..

[23] Zhong V.W., Allen N.B., Greenland P., Carnethon M.R., Ning H., Wilkins J.T., Lloyd-Jones D.M., Van Horn L. (2021). Protein Foods from Animal Sources, Incident Cardiovascular Disease and All-Cause Mortality: A Substitution Analysis. Int. J. Epidemiol..

[24] Jiang H., Wang L., Wang D., Yan N., Li C., Wu M., Wang F., Mi B., Chen F., Jia W. (2022). Omega-3 Polyunsaturated Fatty Acid Biomarkers and Risk of Type 2 Diabetes, Cardiovascular Disease, Cancer, and Mortality. Clin. Nutr..

[25] De Souza R.J., Mente A., Maroleanu A., Cozma A.I., Ha V., Kishibe T., Uleryk E., Budylowski P., Schünemann H., Beyene J. (2015). Intake of Saturated and Trans Unsaturated Fatty Acids and Risk of All Cause Mortality, Cardiovascular Disease, and Type 2 Diabetes: Systematic Review and Meta-Analysis of Observational Studies. BMJ.

[26] Muto M., Ezaki O. (2018). High Dietary Saturated Fat Is Associated with a Low Risk of Intracerebral Hemorrhage and Ischemic Stroke in Japanese but Not in Non-Japanese: A Review and Meta-Analysis of Prospective Cohort Studies. J. Atheroscler. Thromb..

[27] Mazidi M., Mikhailidis D.P., Sattar N., Toth P.P., Judd S., Blaha M.J., Hernandez A.V., Penson P.E., Banach M. (2020). Association of Types of Dietary Fats and All-Cause and Cause-Specific Mortality: A Prospective Cohort Study and Meta-Analysis of Prospective Studies with 1,164,029 Participants. Clin. Nutr..

[28] Jakobsen M.U., O’Reilly E.J., Heitmann B.L., Pereira M.A., Bälter K., Fraser G.E., Goldbourt U., Hallmans G., Knekt P., Liu S. (2009). Major Types of Dietary Fat and Risk of Coronary Heart Disease: A Pooled Analysis of 11 Cohort Studies. Am. J. Clin. Nutr..

